# Supplementary material for: Self-promoted electroactive biomimetic mineralized scaffolds for bacteria-infected bone regeneration
Source: Nat Commun. 2023 Oct 31;14:6963. doi: 10.1038/s41467-023-42598-4 (PMC10618168; doi:10.1038/s41467-023-42598-4)
Supplement: Supplementary file 1 — Supplementary Information [file 41467_2023_42598_MOESM1_ESM.pdf]

Self-promoted electroactive biomimetic mineralized scaffolds for bacteria-infected bone regeneration

Zixin Li[1,2,3,7], Danqing He[1,4,7], Bowen Guo[2], Zekun Wang[2], Huajie Yu[5], Yu Wang[1,4], Shanshan Jin[1,4], Min Yu[1,4], Lisha Zhu[1,4], Liyuan Chen[1,4], Chengye Ding[1,4], Xiaolan Wu[1,4], Tianhao Wu[1,4], Shiqiang Gong[6], Jing Mao[6], Yanheng Zhou[1,4], Dan Luo[2], Yan Liu[1,4]

[1]Laboratory of Biomimetic Nanomaterials, Department of Orthodontics, Peking University School and Hospital of Stomatology, Beijing 100081, PR China

[2]Beijing Institute of Nanoenergy and Nanosystems, Chinese Academy of Sciences, Beijing 101400, PR China

[3]Department of Stomatology, Peking University Peoples Hospital, Beijing 100044, PR China

[4]National Center for Stomatology & National Clinical Research Center for Oral Diseases &National Engineering Research Center of Oral Biomaterials and Digital Medical Devices & Beijing Key Laboratory of Digital Stomatology & Research Center of Engineering and Technology for Computerized Dentistry Ministry of Health & NMPA Key Laboratory for Dental Materials & Translational Research Center for Orocraniofacial Stem Cells and Systemic Health, Beijing 100081, PR China

[5]Fourth Clinical Division, Peking University School and Hospital of Stomatology, Beijing 100081, PR China

[6]Center of Stomatology, Tongji Hospital, Tongji Medical College, Hubei Province Key Laboratory of Oral and Maxillofacial Development and Regeneration, Huazhong University of Science and Technology, Wuhan 430030, PR China

[7]These authors contributed equally to this work: Zixin Li, Danqing He.

Correspondence should be addressed to D.L. (E-mail: luodan@binn.cas.cn) and Y.L. (Email: orthoyan@bjmu.edu.cn).

Supplementary figures

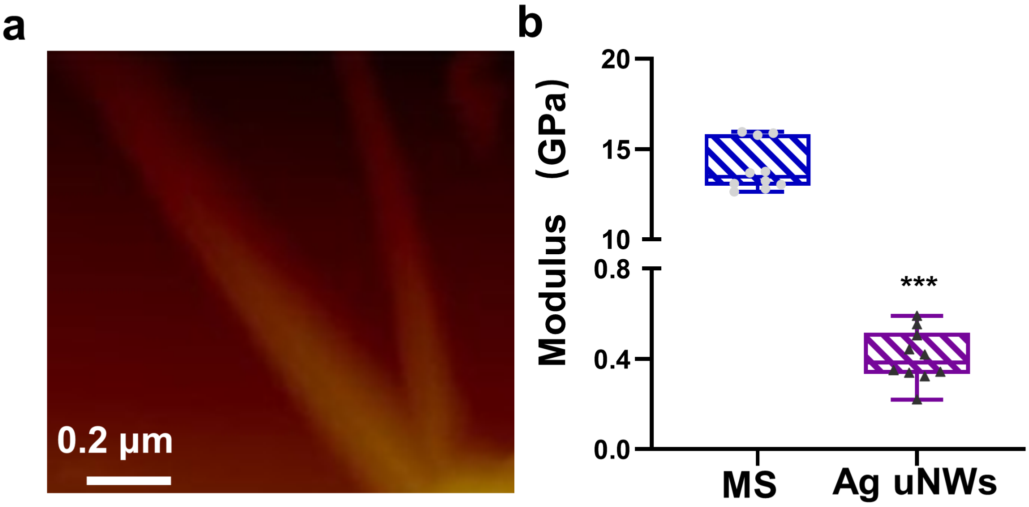

Supplementary Fig. 1. a, A representative AFM image of Ag uNWs. **b,** Young's modulus of MS and Ag uNWs (n = 10 biologically independent samples, by two-tailed Student's t-test: *** $P < 0.001$ versus MS. Each box plot represents the minimum, first quartile, median, third quartile, and maximum of 10 values; the whiskers were drawn down to the minimum and up to the maximum). Source data and exact P values are provided in the Source Data file.

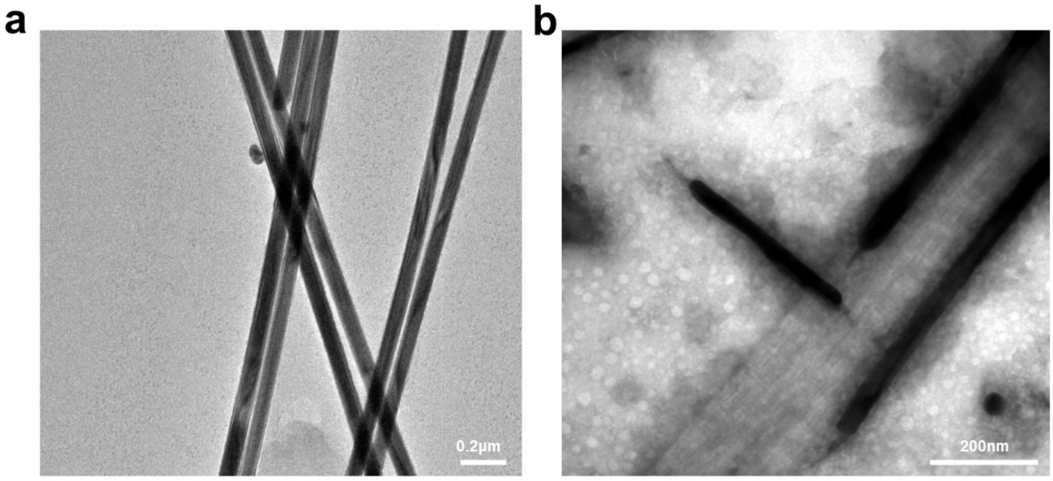

Supplementary Fig. 2. Micro-nano topology of thick AgNWs and composites of thick AgNWs/mineralized collagen. **a,** TEM image of thick AgNWs with diameter of 53.63 ± 6.17 nm. **b,** TEM image showing thick AgNW separation from the collagen phase.

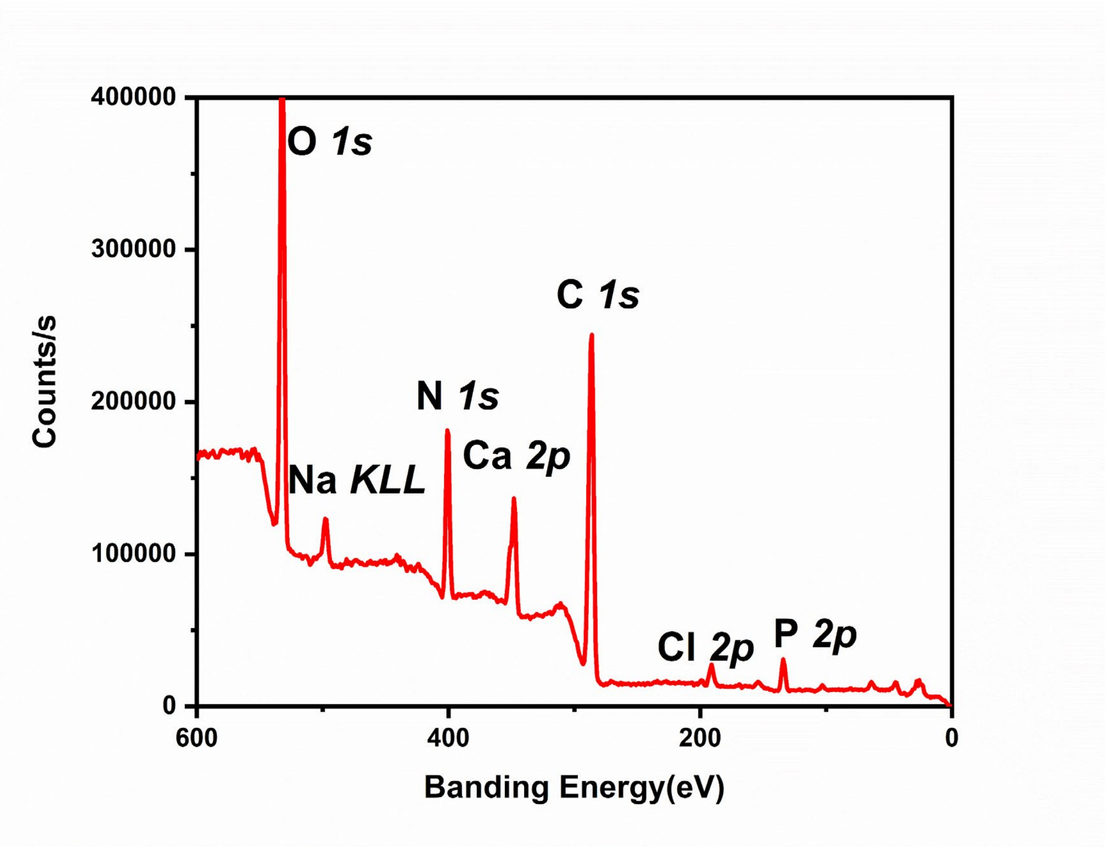

Supplementary Fig. 3. XPS measurements of MS.

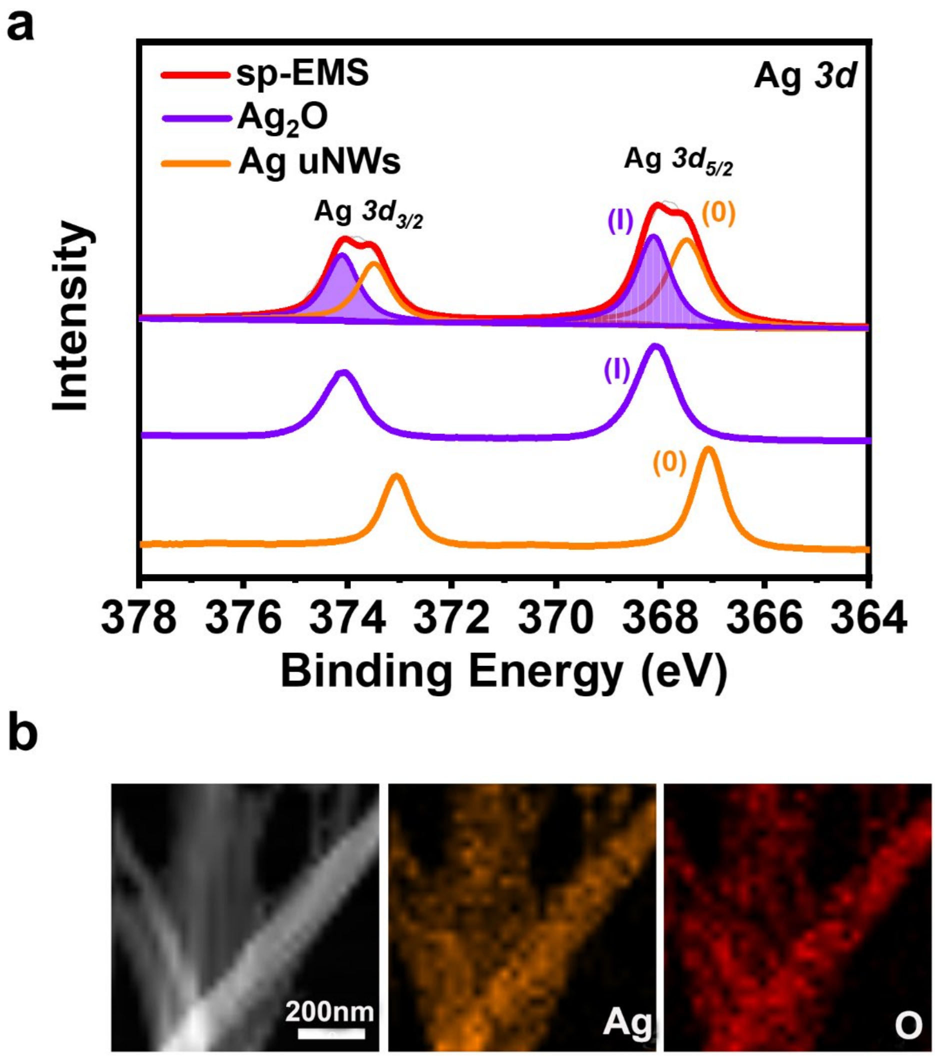

Supplementary Fig. 4. XPS spectra and EDS mapping of the sp-EMS after electrochemical corrosion. **a,** Ag *3d* XPS spectra of sp-EMS, Ag uNWs, and Ag_2O, respectively. After 1 day of corrosion, high resolution Ag 3d spectrum of sp-EMS could be fitted with spin-orbit split $3d_{5/2}$ and $3d_{3/2}$, corresponding to oxidation state of Ag-O bonds and zero valent Ag, respectively. The results confirm that the electrochemical corrosion products of Ag uNWs could coordinate with the carboxyl groups enriched in collagen. **b,** EDS mapping of sp-EMS after electrochemical corrosion. Similarly, EDS mapping also confirmed the presence of a large number of coordinated oxygen atoms on the surface of Ag uNWs after 1 day of corrosion. The coassembled structure of Ag uNWs with mineralized collagen fibrils in sp-EMS and the formation of Ag-O coordination bonds are essential to accelerate the electrochemical corrosion of Ag uNWs, thereby achieving self-promoted electroactivity.

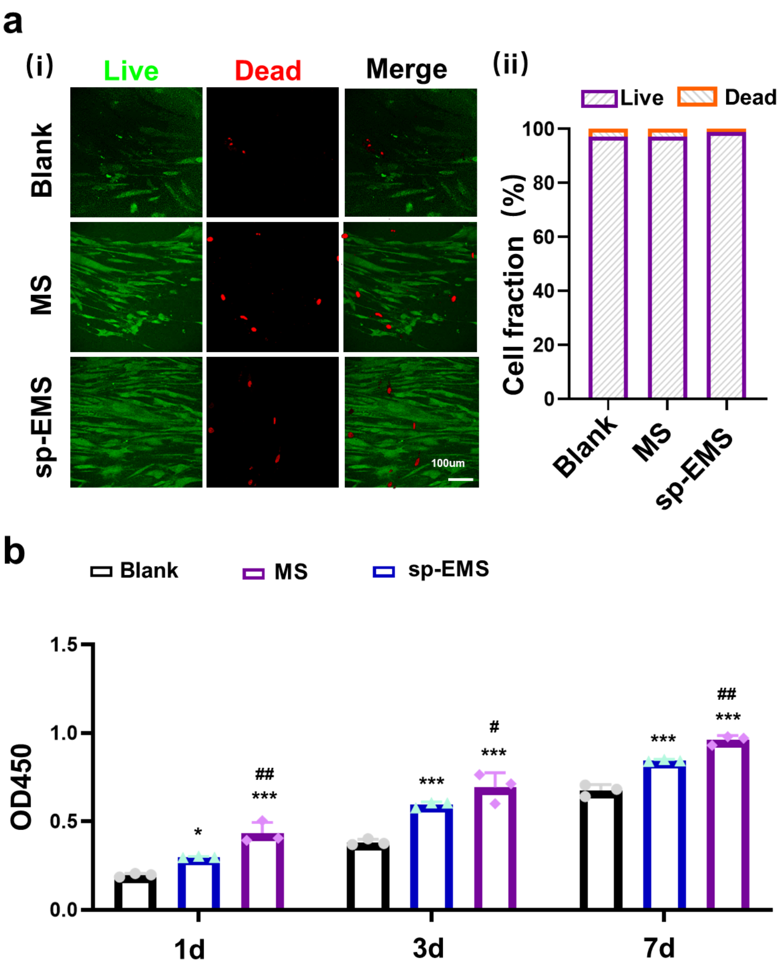

Supplementary Fig. 5. The biocompatibility of different scaffolds. **a,** (i) Representative live/dead staining of BMSCs seeded on the 6-well plate (blank), MS, and sp-EMS for 1 day. (ii) Semiquantification of (i) (n = 3 biologically independent samples, by one-way ANOVA with Tukey's post hoc test: $P > 0.05$ versus blank and MS. Data are presented as means). **b**, CCK-8 assay of BMSCs seeded on different scaffolds for 1 day, 3 days, and 7 days (n = 3 biologically independent samples, by one-way ANOVA with Tukey's post hoc test: * $P < 0.05$, *** $P < 0.001$ versus blank; # $P <$ 0.05, ### $P < 0.01$ versus MS). Data are presented as means ± SD. Source data and exact P values are provided in the Source Data file.

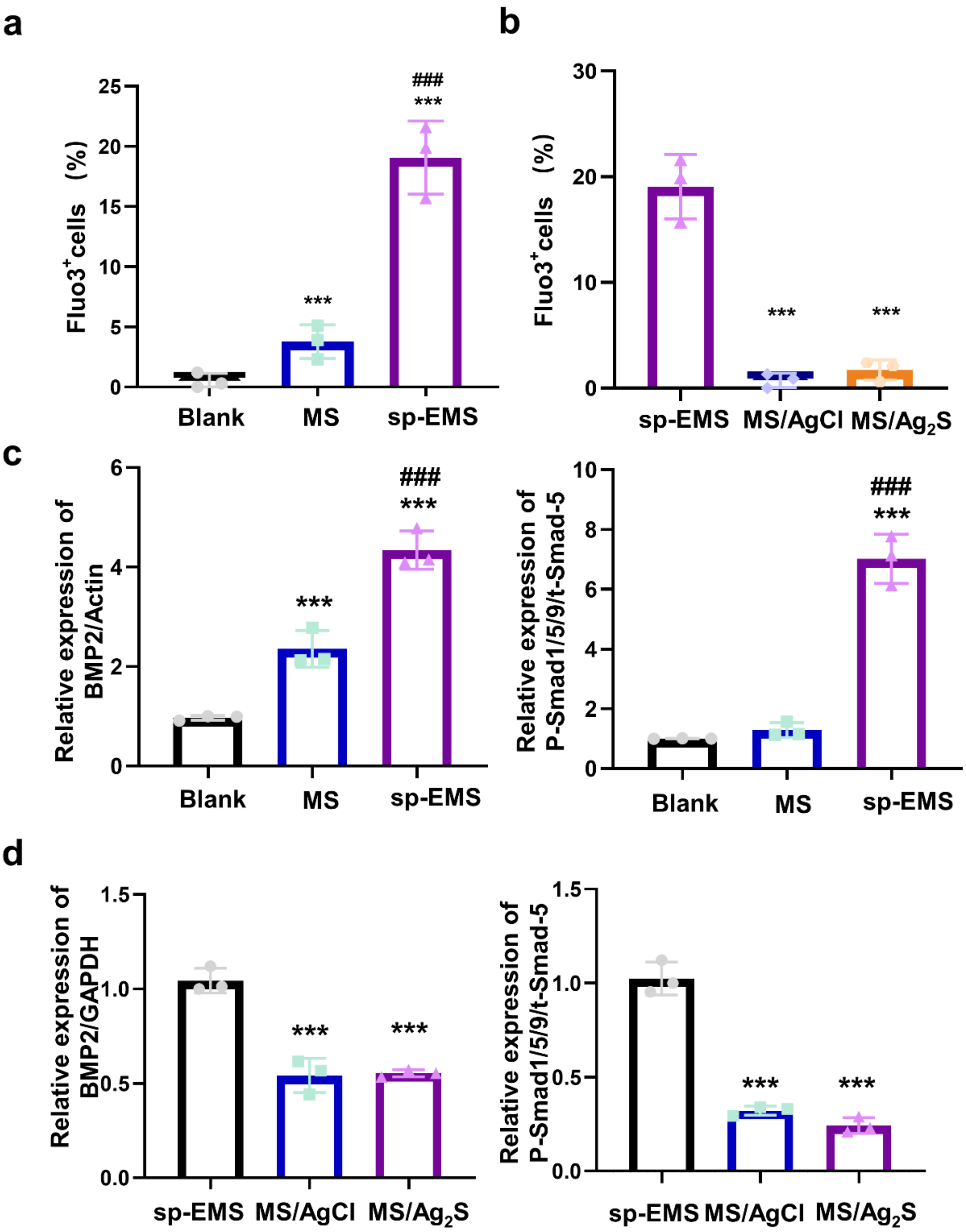

Supplementary Fig. 6. Semiquantification in Fig. 2. **a,** Semiquantification of Fluo-3$^+$ cells seeded on the 6-well plate (blank), MS, and sp-EMS (n = 3 biologically independent samples, by one-way ANOVA with Tukey's post hoc test: *** $P < 0.001$ versus blank; ### $P < 0.001$ versus MS). **b,** Semiquantification of Fluo-3$^+$ cells seeded on the sp-EMS, MS/AgCl, and MS/Ag_2S (n = 3 biologically independent samples, by one-way ANOVA with Tukey's post hoc test: *** $P < 0.001$ versus sp-EMS). **c,** Semiquantification of the expressions of BMP2, p-Smad1/5/9, and t-Smad5 in BMSCs cultured on the 6-well plate, MS, and sp-EMS for 7 days (n = 3 biologically independent

samples, by one-way ANOVA with Tukey's post hoc test: *** $P < 0.001$ versus blank; ### $P < 0.001$ versus MS). **d,** Semiquantification of the expressions of BMP2, p-Smad1/5/9, and t-Smad5 in BMSCs cultured on the sp-EMS, MS/AgCl, and MS/Ag_2S for 7 days ($n = 3$ biologically independent samples, by one-way ANOVA with Tukey's post hoc test: *** $P < 0.001$ versus sp-EMS). Data are presented as means ± SD. Source data and exact P values are provided in the Source Data file.

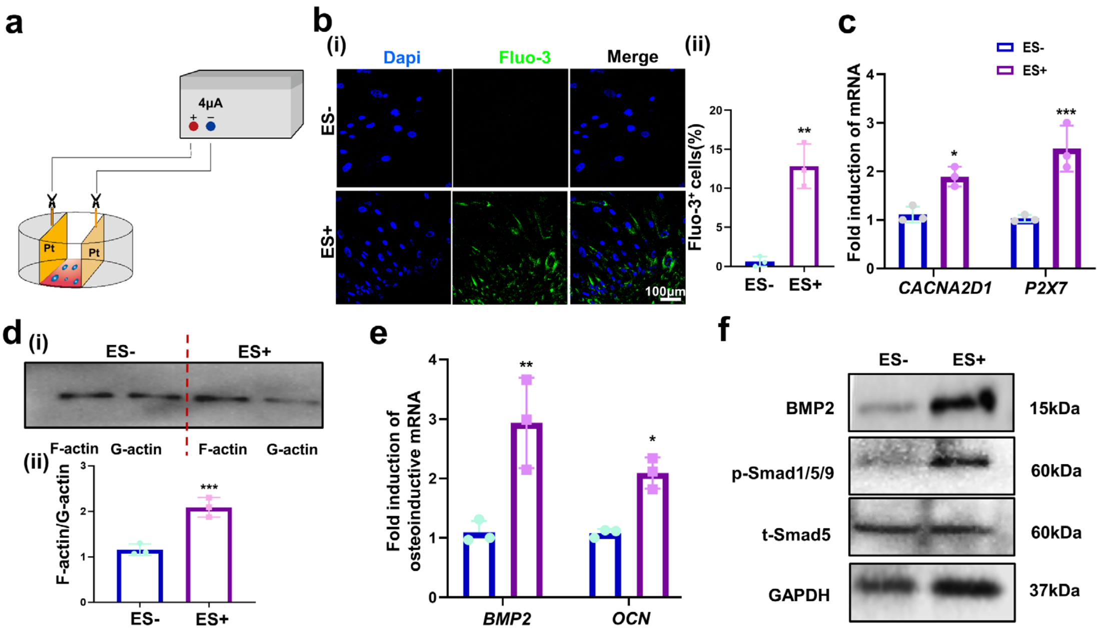

Supplementary Fig. 7. Electrical stimulation induces osteogenic differentiation of human BMSCs *in vitro.* **a,** Schematic of exogenous electrical stimulation to BMSCs. **b,** (i) Representative immunofluorescence staining of Fluo-3 after 48 h of electrical stimulation. (ii) Semiquantification of Fluo-3$^+$ cells in (i) ($n = 3$ biologically independent samples, by two-tailed Student's t-test: ** $P < 0.01$ versus ES-). **c,** Relative mRNA expressions of calcium channel (*CACNA2D1*) and purinoceptors (*P2X7*) in BMSCs after 48 h of electrical stimulation ($n = 3$ biologically independent samples, by two-tailed Student's t-test: * $P < 0.05$, *** $P < 0.001$ versus ES-). **d,** (i) Western blotting of actin in BMSCs by differential ultracentrifugation after 48 h of electrical stimulation. (ii) Ratio of F-actin to G-actin from densitometry of actin bands in (i) ($n = 3$ biologically independent samples, by two-tailed Student's t-test: *** $P < 0.001$ versus ES-). **e,** Relative mRNA expressions of the expressions of osteogenic differentiation markers *BMP2* and *OCN* in BMSCs after 48 h of electrical stimulation ($n = 3$ biologically independent samples, by two-tailed Student's t-test: * $P < 0.05$, ** $P < 0.01$ versus ES-).

f, Western blotting of the expressions of BMP2, p-Smad1/5/9, and t-Smad5 in BMSCs after 48 h of electrical stimulation. GAPDH served as an internal control for equal loading (n = 3 biologically independent samples). ES-: without electrical stimulation. ES+: electrical stimulation. Data are presented as means ± SD. Source data and exact P values are provided in the Source Data file.

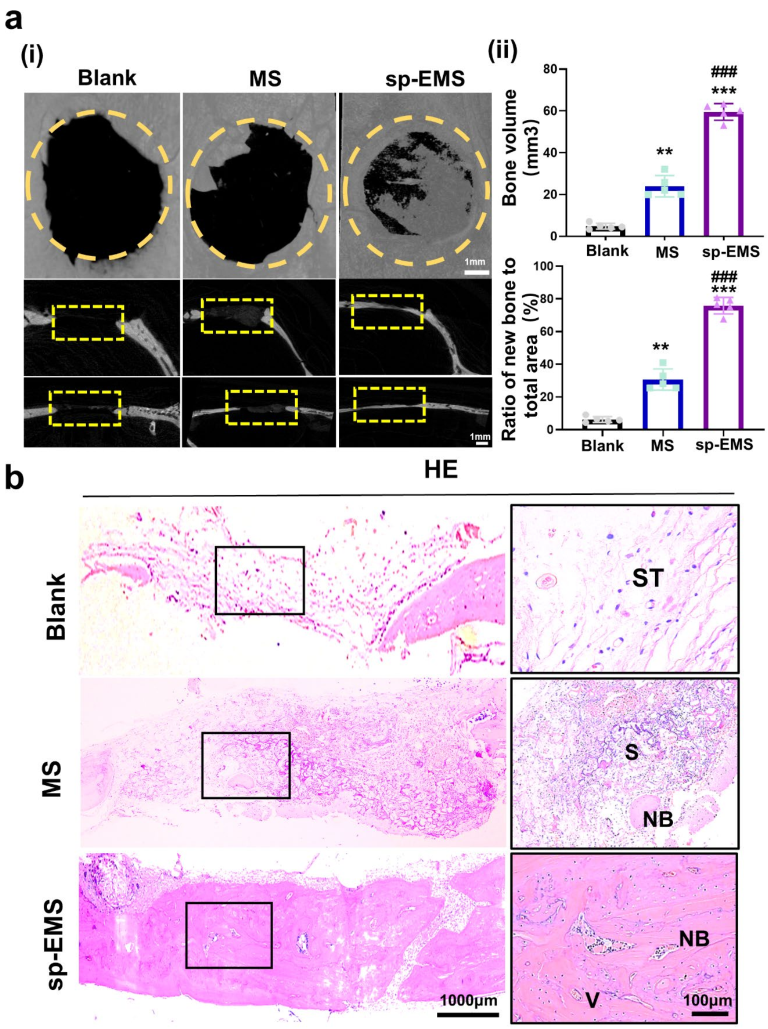

Supplementary Fig. 8. Bone regeneration in rat noninfected calvarial bone defects after 8-week implantation. **a,** (i) Representative μCT images of rat noninfected calvarial defect repair in the blank, MS, and sp-EMS groups after 8-week implantation. Yellow circle displays defect boundary. (ii) Semiquantification of the bone volume and the ratio of new bone to total areas in (i) (n = 5 rat critical-sized noninfected calvarial defects per group, by one-way ANOVA with Tukey's post hoc test: *** $P < 0.001$ versus blank; ### $P < 0.001$ versus MS). **b,** Representative HE staining images of the engineered bones after 8-week implantation. ST: soft tissue, NB: new bone, V: vessel, S: scaffolds. Data are presented as means ± SD. Source data and exact P values are provided in the Source Data file.

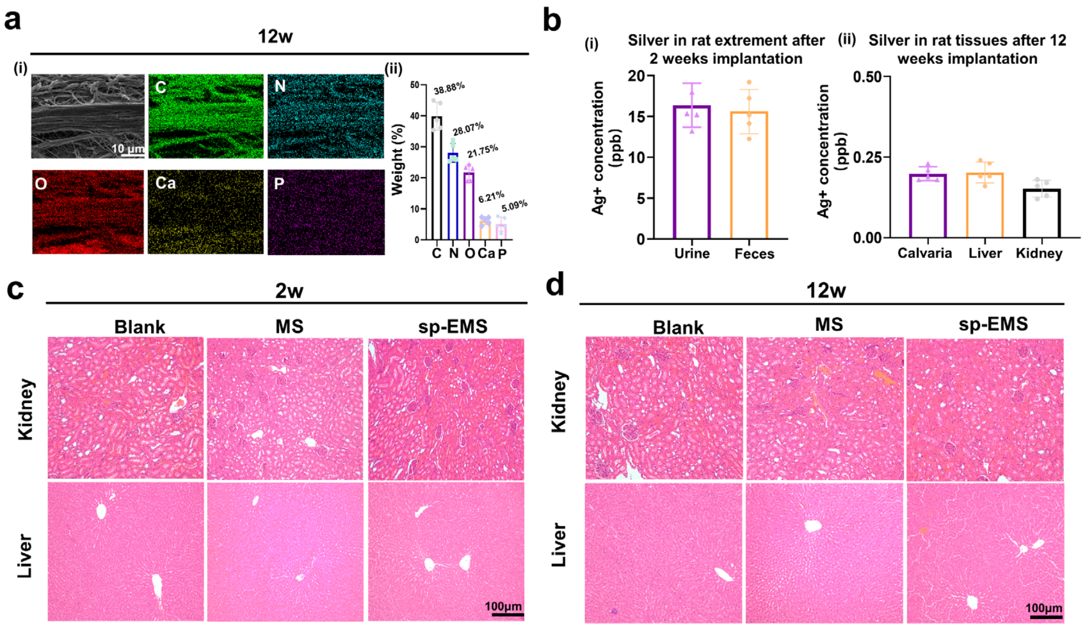

Supplementary Fig. 9. The biosafety of the sp-EMS *in vivo*. **a,** (i) A representative SEM image and mapping of newly-formed calvaria after implantation of the sp-EMS for 12 weeks. (ii) Weight content of C, N, O, Ca, and P in newly-formed calvaria (n = 5 rat critical-sized noninfected calvarial defects per group). **b,** (i) The ICP-OES test of silver concentration in rat urine and feces after implantation of the sp-EMS for 2 weeks. (ii) The ICP-OES test of silver concentration in calvaria, liver, and kidney after implantation of the sp-EMS for 12 weeks (n = 5 rats per group). **c,d,** Representative HE staining images of main metabolic organs (liver and kidney) after the implantation of different scaffolds at 2 weeks (c) and 12 weeks (d). Data are presented as means ± SD. Source data are provided in the Source Data file.

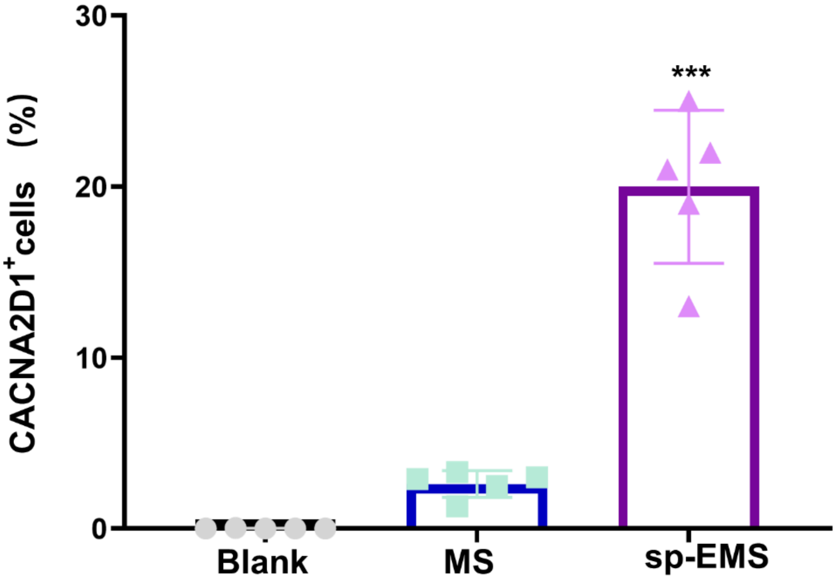

Supplementary Fig. 10. Semiquantification of CACNA2D1 positive cells in rat noninfected calvarial bone defect areas after 2-week implantation in Fig. 3 (n = 5 rat critical-sized noninfected calvarial defects per group, by one-way ANOVA with Tukey's post hoc test: *** $P < 0.001$ versus blank and MS). Data are presented as means ± SD. Source data and exact P values are provided in the Source Data file.

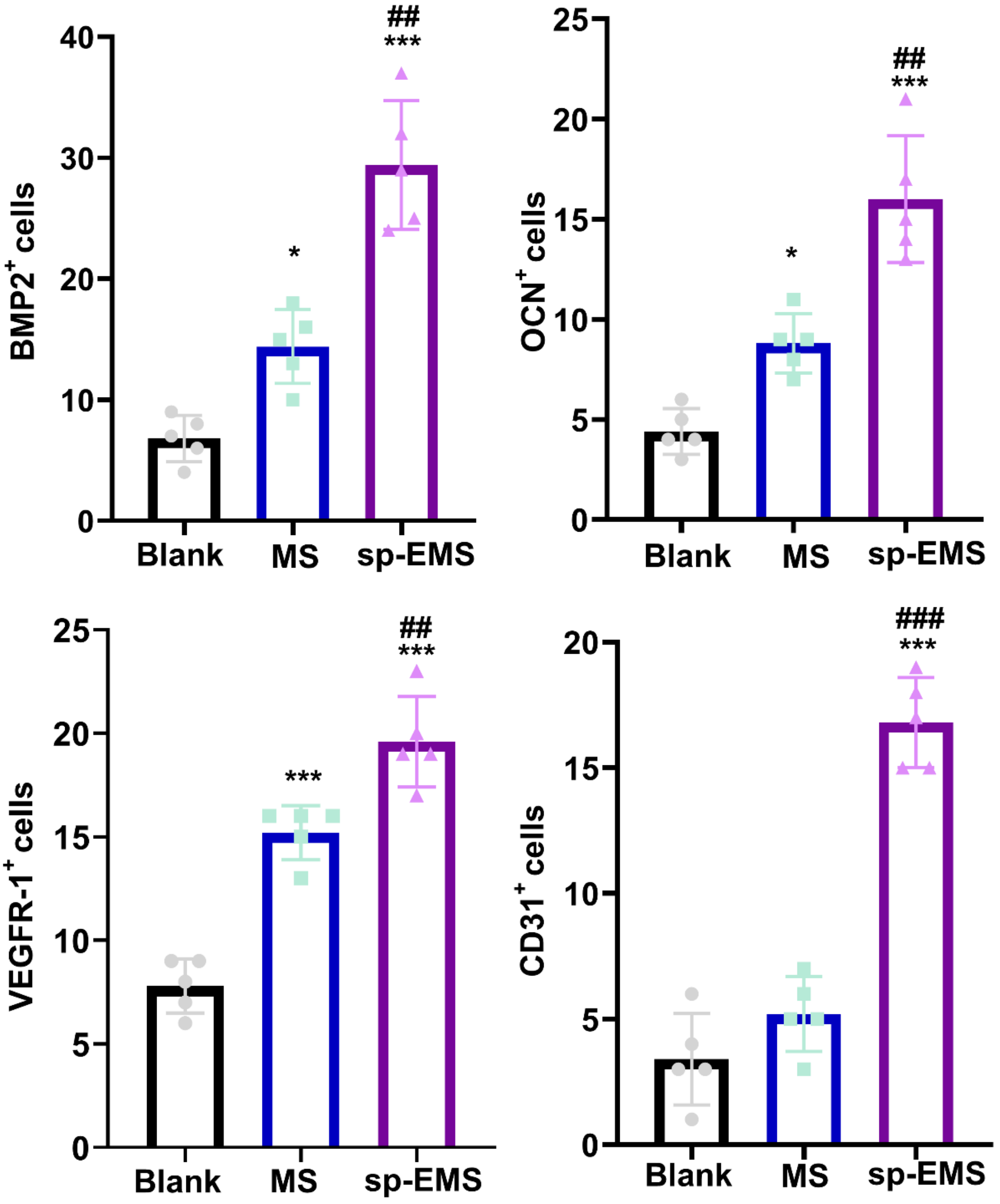

Supplementary Fig. 11. Semiquantification of BMP2, OCN, VEGFR-1, and CD31 positive cells in rat noninfected calvarial bone defect areas after 8-week implantation in Fig. 3 (*n* = 5 rat critical-sized noninfected calvarial defects per group, by one-way ANOVA with Tukey's post hoc test: * $P < 0.05$, *** $P < 0.001$ versus blank; ## $P < 0.01$, ### $P < 0.001$ versus MS). Data are presented as means ± SD. Source data and exact *P* values are provided in the Source Data file.

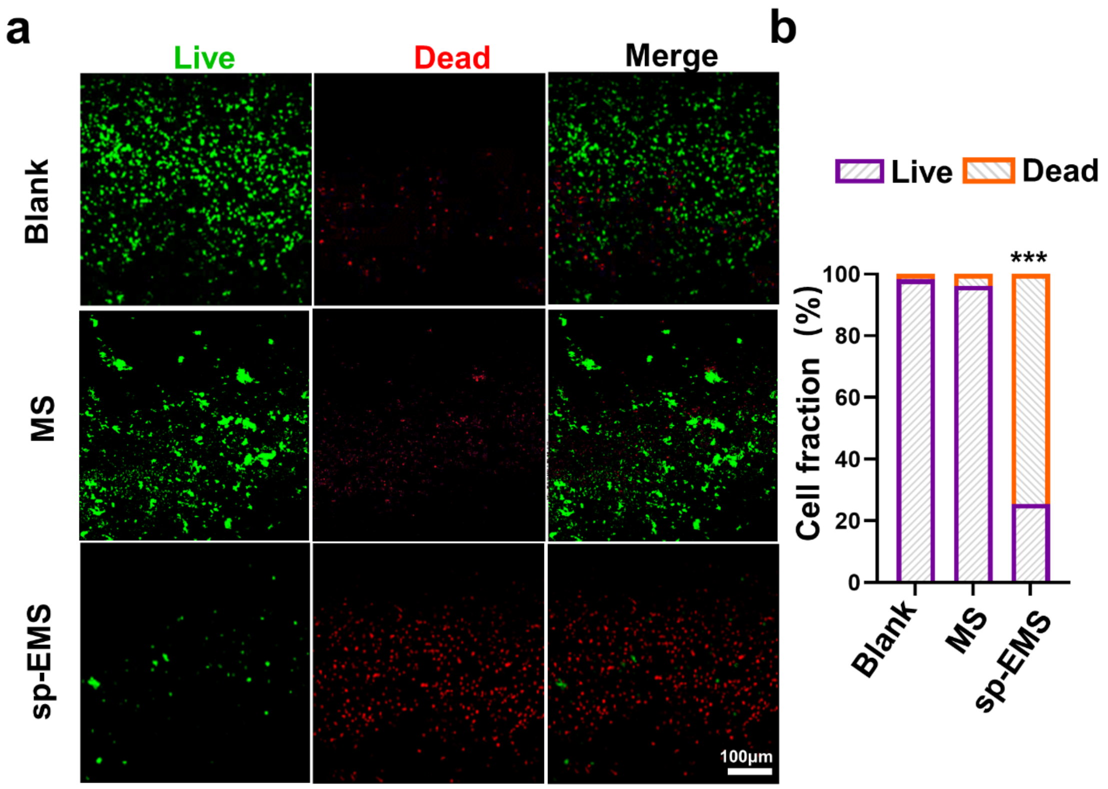

Supplementary Fig. 12. The antibacterial properties of the sp-EMS *in vitro*. **a,** Representative live/dead staining for *S. aureus* seeded on the 6-well plate (blank), MS, and sp-EMS for 1 day. **b,** Semiquantification of live/dead staining (n = 3 biologically independent samples, by one-way ANOVA with Tukey's post hoc test: *** $P < 0.001$ versus blank and MS). Data are presented as means. Source data and exact P values are provided in the Source Data file.

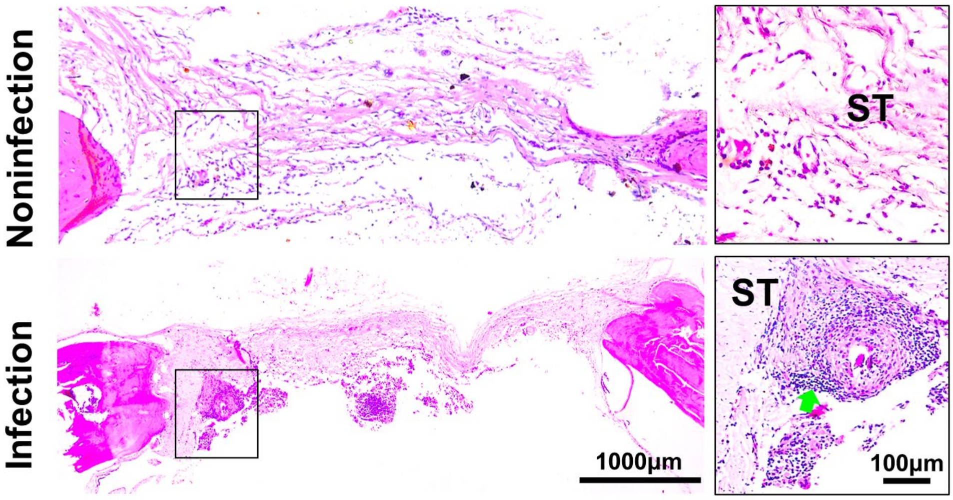

Supplementary Fig. 13. Establishment of a rat calvarial infected bone defect model. Compared to the rat calvarial noninfected bone defect model, the infected bone defect model without any implants exhibited a large accumulation of inflammatory cells 2 weeks after surgery, confirming the presence of infection. ST: soft tissue. Green arrow displays inflammatory cells.

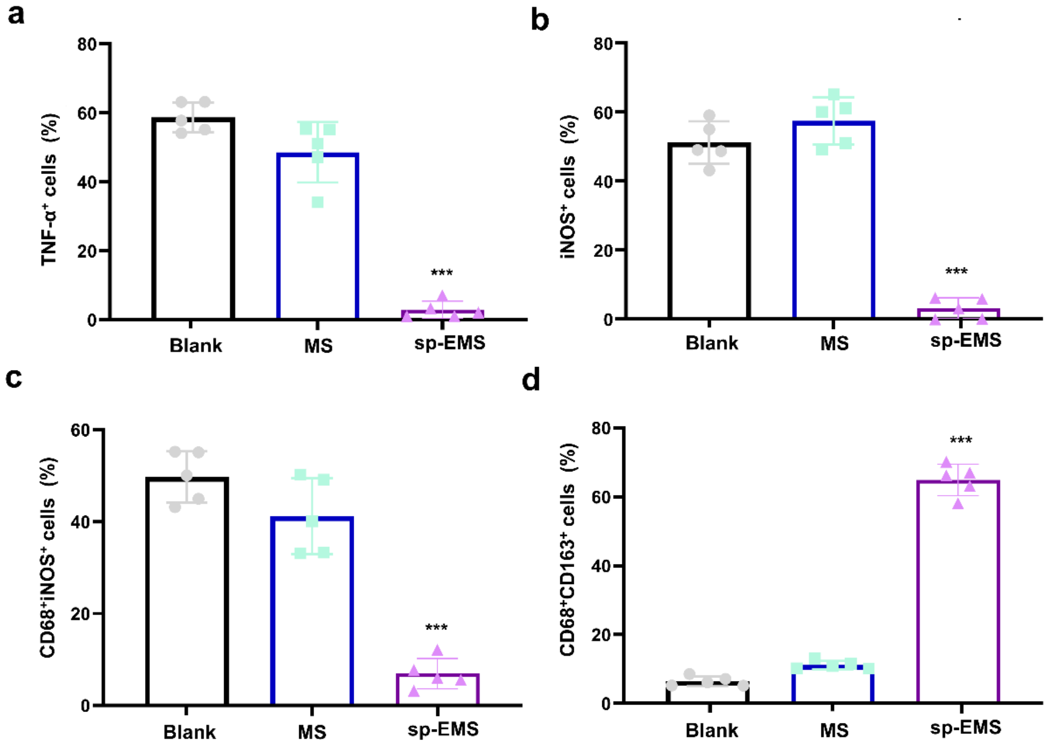

Supplementary Fig. 14. Semiquantification of TNF-α^+ (a), iNOS$^+$ (b), CD68$^+$iNOS$^+$ (c), and CD68$^+$CD163$^+$ (d) cells in rat infected calvarial bone defect areas after 2-week implantation in Fig. 5 (n = 5 rat critical-sized infected calvarial defects per group, by one-way ANOVA with Tukey's post hoc test: *** $P < 0.001$ versus blank and MS). Data are presented as means ± SD. Source data and exact P values are provided in the Source Data file.

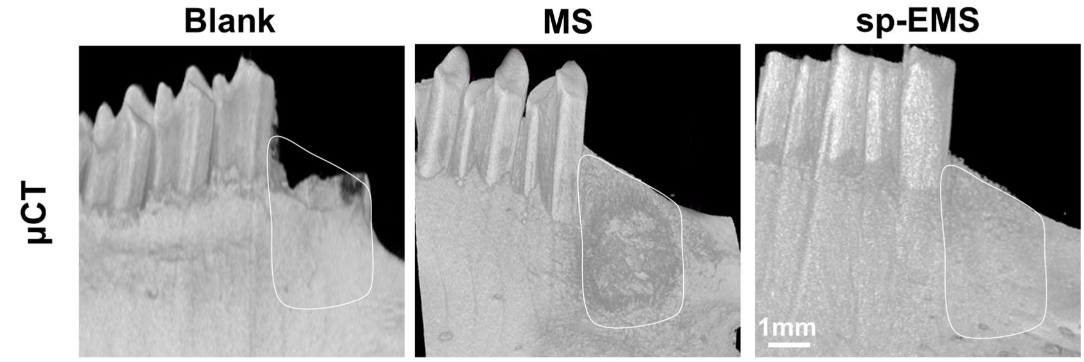

Supplementary Fig. 15. Representative μCT 3D reconstruction images of the rabbit engineered bones in the blank, MS, and sp-EMS groups after 8 weeks of implantation. White lines display the defect boundaries.

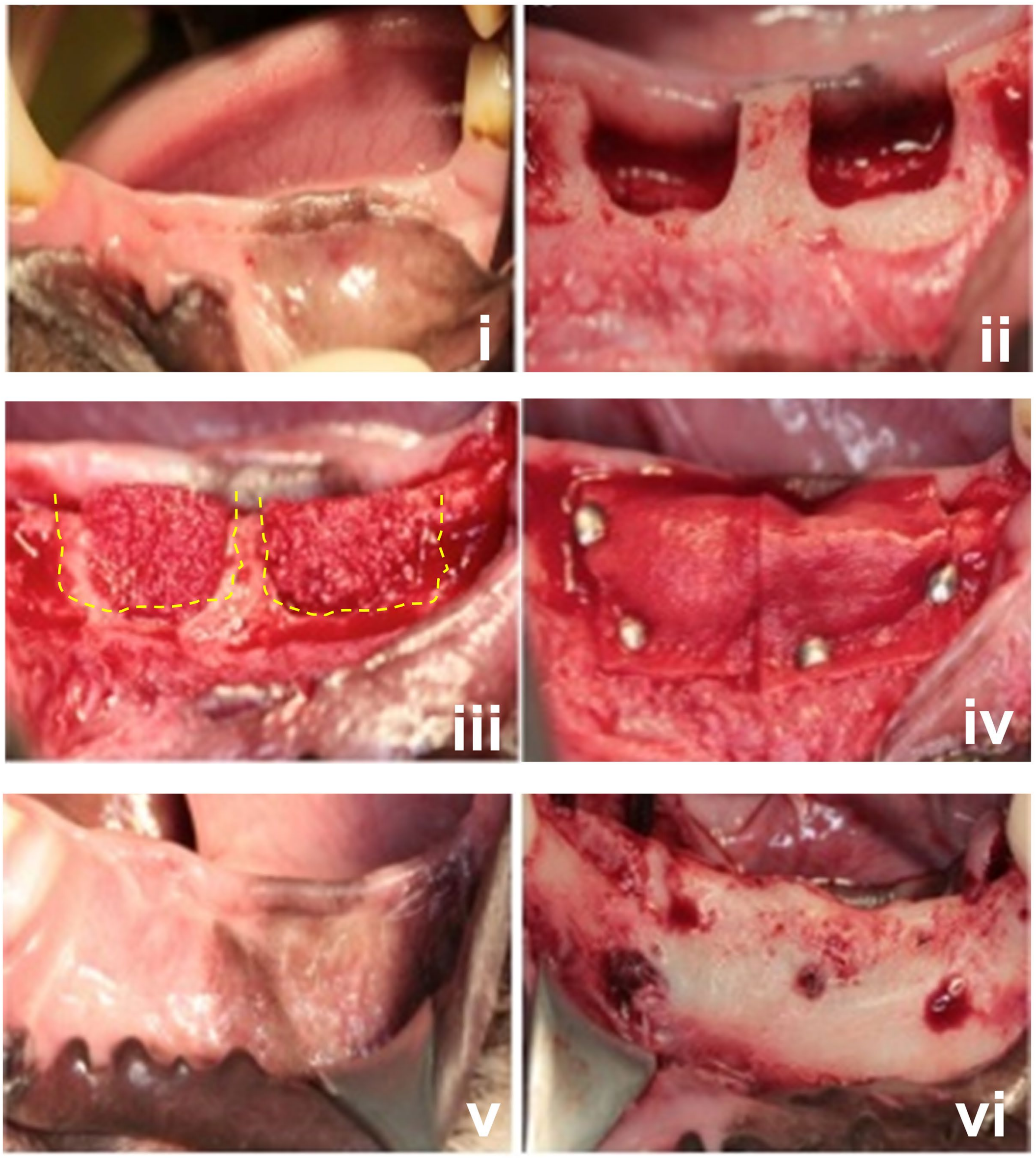

Supplementary Fig. 16. Gross morphology of the beagle mandibular vertical bone regeneration. (i) All premolars and first molars in mandibles of adult male beagle dogs were extracted. (ii) After healing for 3 months, two vertical bone defects with 10 mm×10 mm×8 mm were created at each mandible. (iii) Different scaffolds were placed into the bone defect areas. (iv) The vertical bone defects were covered by biological membranes. (v) Lateral view of the surgical site after 12-week healing. (vi) Profile of newly formed bone after mucosal stripping.

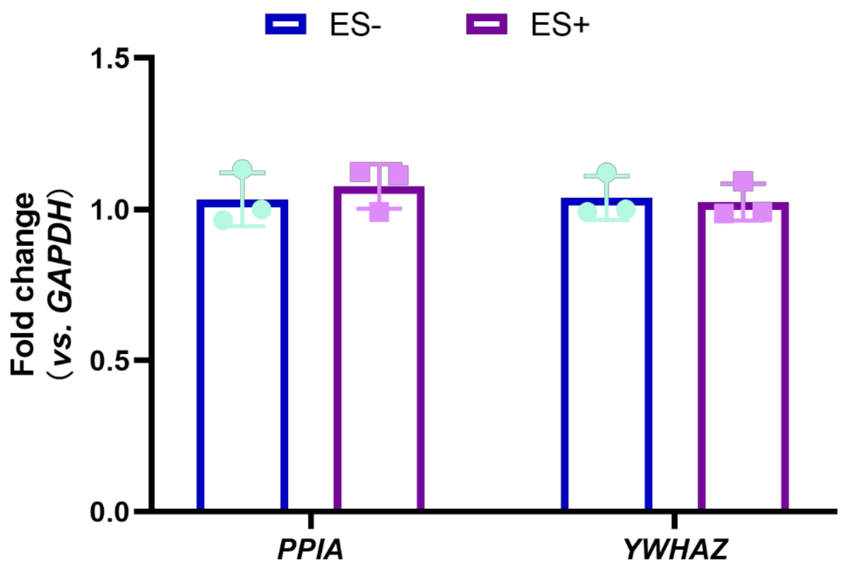

Supplementary Fig. 17. Relative mRNA expression levels of *PPIA* and *YWHAZ* normalized to *GAPDH* in BMSCs after electrical stimulation for 48 h ($n = 3$ biologically independent samples, by two-tailed Student's t-test). Data are presented as means ± SD. Source data and exact P values are provided in the Source Data file.

Supplementary Table 1. List of chemicals and recombinant proteins used in the study.

**Chemicals and peptides**		
Penicillin-Streptomycin	Thermo Fisher Scientific	Cat#15070063
Trypsin-EDTA	Hyclone	Cat#SH30042.01
TRIzol Reagent	Thermo Fisher Scientific	Cat#15596026
Collagen I, Rat Tail	Corning	Cat#54236
Mounting Medium with DAPI	ZSGB-BIO	Cat#ZLI-9557
1-ethyl3-(3-dimehylaminopropyl) carbodiimide hydrochloride	Sigma-Aldrich	Cat#25952-53-8
RIPA Buffer	Thermo Fisher Scientific	Cat#89900
L-Glutamine	Thermo Fisher Scientific	Cat#25030081
Fetal bovine serum（FBS）	Thermo Fisher Scientific	Cat#10099-141
SYBR Green Supermix	Thermo Fisher Scientific	Cat#4385612
Dexamethasone	Sigma-Aldrich	Cat#D8893
TBS	Solarbio	T1080
Tween	Sigma-Aldrich	P9416
Bovine serum albumin (BSA)	Solarbio	Cat#A8010
Sodium phosphotungstate	Aladdin	Cat#51312-42-6
Poly- (α, β)-DL-aspartic acid sodium salt	Sigma-Aldrich	Cat#94525-01-6
N-Hydroxysuccinimide	Sigma-Aldrich	Cat#6066-82-6
Lipoteichoic acid from *Staphylococcus aureus*	Sigma-Aldrich	Cat#56411-57-5
LB Broth	Sigma-Aldrich	Cat#L7275
Bio-Oss®	Geistlich Pharma AG	Cat#20141
2′,7′-Dichlorofluorescin diacetate	Solarbio	Cat#4091-99-0
Fluo 3-AM	Solarbio	Cat#121714-22-5
**Critical commercial assays**		
ReverTra Ace qPCR RT Kit	TOYOBO	Cat#FSQ-101
Masson’s Trichrome Stain Kit	Solarbio	Cat#G1340
G-Actin/F-Actin In Vivo Assay Biochem Kit	Cytoskeleton	Cat#BK037
Pierce BCA protein assay Kit	Thermo Fisher Scientific	Cat#23225
Protease/Phosphatase Inhibitor	Thermo Fisher Scientific	Cat#87786

Cocktail		
Enhanced Chemiluminescence Western Blotting Detection Kit	Thermo Fisher Scientific	Cat#34577
DAB peroxidase substrate kit	ZSGB-BIO	ZLI-9017
Cell Counting Kit-8	Solarbio	Cat#1210
Calcein-AM/PI Live/Dead Stain Kit	Solarbio	Cat#1630

Supplementary Table 2. List of primers used in the study.

Gene	Assay	Forward (5'-3')	Reverse (5'-3')
hGAPDH	qRT-PCR	TCGGAGTCAACGGATTTGGT	TTGCCATGGGTGGAATCATA
hP2X7	qRT-PCR	TGCTCTCTTGAACAGTGCCG	CCTGGCAGGATGTTTCTCGT
hCACNA2D1	qRT-PCR	GACTGACCAACACCACTCTTCAC	ATCGTACCTCAGCTCCTTCC
hBMP2	qRT-PCR	CAGAGACCCACCCCCAGCA	CTGTTTGTGTTTGGCTTGAC
hOCN	qRT-PCR	CCCAGGCGCTACCTGTATCAA	GGTCAGCCAACTCGTCACAGTC
hTNF-α	qRT-PCR	TACCGGCATTATTGGAGTGA	GTGTTCTGTTTCTCCTGGCA
hiNOS	qRT-PCR	GAGCTTCTACCTCAAGCTATC	CCTGATGTTGCCATTGTTGGT
hPPIA	qRT-PCR	TGCTGGACCCAACACAAATG	AACACCACATGCTTGCCATC
hYWHAZ	qRT-PCR	CGAAGCTGAAGCAGGAGAAG	TTTGTGGGACAGCATGGATG

Supplementary Table 3. List of reagents or resources used in the study.

REAGENT or RESOURCE	SOURCE	IDENTIFIER
**Antibodies**		
Rabbit polyclonal anti-CACNA2D1	Abcam	Cat# AB238110
Rabbit monoclonal anti-BMP2	Abcam	Cat#AB214821
Rabbit polyclonal anti-OCN	Abcam	Cat# AB93876
Rabbit polyclonal anti-VEGFR-1	Abcam	Cat# AB2350
Rabbit polyclonal anti-CD31	Abcam	Cat#AB32457
Rabbit monoclonal anti- TNF-α	Abcam	Cat#AB183218
Rabbit polyclonal anti-CD68	Proteintech	Cat#28058-1-AP
Rabbit monoclonal anti-p-Smad1/5/9	Cell signaling technology	Cat#13820
Rabbit monoclonal anti-Smad5	Cell signaling technology	Cat#12534
Mouse monoclonal anti-CD163	Abcam	Cat#AB156769
Mouse monoclonal anti-iNOS	Abcam	Cat#AB178945
Mouse monoclonal anti-GADPH	Proteintech	Cat#60004-1-lg
Mouse monoclonal anti-Actin	ZSGB-BIO	Cat#TA-09
HRP-linked anti-rabbit IgG	ZSGB-BIO	Cat#ZB-2305
HRP-linked anti-mouse IgG	ZSGB-BIO	Cat#ZB-2301
FITC-labeled goat anti-rabbit IgG(H+L)	ZSGB-BIO	Cat#ZF-0311
Rhodamine-labeled goat anti-mouse IgG(H+L)	ZSGB-BIO	Cat#ZF-0313
Horseradish enzyme labeled goat anti-rabbit IgG	ZSGB-BIO	Cat#PV-9001

Supplementary Table 4. List of animals used in the study.

Defects	Sample size	Time points	Groups
Rat critical-sized noninfected calvarial defects	45	2 weeks<br>8 weeks<br>12 weeks	Blank (n = 5 at each time point)<br>MS (n = 5 at each time point)<br>sp-EMS (n = 5 at each time point)
Rat critical-sized infected calvarial defects	30	2 weeks<br>12 weeks	Blank (n = 5 at each time point)<br>MS (n = 5 at each time point)<br>sp-EMS (n = 5 at each time point)
Rabbit open bone defects	5 (bilateral alveolar open defects in one rabbit and totally 10 defects)	8 weeks	Blank (n = 3)<br>MS (n = 3)<br>sp-EMS (n = 3)
Dog vertical bone defects	3 (two bone defects at each side and four defects in one dog and totally 12 defects)	12 weeks	Blank (n = 3)<br>Bio-Oss (n = 3)<br>MS (n = 3)<br>sp-EMS (n = 3)

Supplementary Table 5. Software and Algorithms.

μCT Evaluation CTAn software (version 1.15)	https://www.blue-scientific.com/bruker-micro-ct-software/
CTvox 3.2	https://www.blue-scientific.com/bruker-micro-ct-software/
Dataviewer 1.5	https://www.blue-scientific.com/bruker-micro-ct-software/
NRecon 1.6	https://www.blue-scientific.com/bruker-micro-ct-software/
Graph Pad Prism 8	https://www.graphpad.com/
Origin 2021	https://www.originlab.com/
Nanoscope analysis 1.9	https://www.bruker.com/
MDI Jade 6.5	https://www.icdd.com/mdi-jade/
LAS X 3.5	https://www.leica-microsystems.com.cn
Image J (v1.53k)	https://imagej.nih.gov
