# Supplementary material for: Self-promoted electroactive biomimetic mineralized scaffolds for bacteria-infected bone regeneration
Source: Nat Commun. 2023 Oct 31;14:6963. doi: 10.1038/s41467-023-42598-4 (PMC10618168; doi:10.1038/s41467-023-42598-4)
Supplement: Supplementary file 3 — Description of additional supplementary files [file 41467_2023_42598_MOESM3_ESM.pdf]

Description of additional supplementary files

Supplementary Movie. 1. Video of electrical measurement in rat critical-sized noninfected calvarial defects.
