# Supplementary material for: Self-promoted electroactive biomimetic mineralized scaffolds for bacteria-infected bone regeneration
Source: Nat Commun. 2023 Oct 31;14:6963. doi: 10.1038/s41467-023-42598-4 (PMC10618168; doi:10.1038/s41467-023-42598-4)
Supplement: Supplementary file 5 — Reporting Summary [file 41467_2023_42598_MOESM5_ESM.pdf]

nature portfolio

Corresponding author(s): Prof. Yan Liu

Last updated by author(s): Sep 20, 2023

Reporting Summary

Nature Portfolio wishes to improve the reproducibility of the work that we publish. This form provides structure for consistency and transparency in reporting. For further information on Nature Portfolio policies, see our Editorial Policies and the Editorial Policy Checklist.

Statistics

For all statistical analyses, confirm that the following items are present in the figure legend, table legend, main text, or Methods section.

n/a	Confirmed	
☐	☒	The exact sample size (*n*) for each experimental group/condition, given as a discrete number and unit of measurement
☐	☒	A statement on whether measurements were taken from distinct samples or whether the same sample was measured repeatedly
☐	☒	The statistical test(s) used AND whether they are one- or two-sided<br>*Only common tests should be described solely by name; describe more complex techniques in the Methods section.*
☐	☒	A description of all covariates tested
☐	☒	A description of any assumptions or corrections, such as tests of normality and adjustment for multiple comparisons
☐	☒	A full description of the statistical parameters including central tendency (e.g. means) or other basic estimates (e.g. regression coefficient) AND variation (e.g. standard deviation) or associated estimates of uncertainty (e.g. confidence intervals)
☐	☒	For null hypothesis testing, the test statistic (e.g. *F*, *t*, *r*) with confidence intervals, effect sizes, degrees of freedom and *P* value noted<br>*Give P values as exact values whenever suitable.*
☒	☐	For Bayesian analysis, information on the choice of priors and Markov chain Monte Carlo settings
☒	☐	For hierarchical and complex designs, identification of the appropriate level for tests and full reporting of outcomes
☒	☐	Estimates of effect sizes (e.g. Cohen's *d*, Pearson's *r*), indicating how they were calculated

Our web collection on statistics for biologists contains articles on many of the points above.

Software and code

Policy information about availability of computer code

Data collection	Nanoscope analysis version 1.9, LAS X version 3.5, and micro-CT Evaluation CTAn software version 1.15 were used. All software and equipments were available in the Methods section and Supplementary Table 5.
Data analysis	Graph Pad Prism Version 8.4.3 were used for statistical analysis.<br>Micro-CT images were analyzed with NRecon version 1.6, CTvox version 3.2, and Dataviewer version 1.5.<br>The XRD results were analyzed with MDI Jade Version 6.5.<br>The XPS and electrochemical measurements results were analyzed with Origin 2021.<br>The AFM results were analyzed with Nanoscope analysis version 1.9.<br>Western blot results were analyzed by using image J version 1.53k.

For manuscripts utilizing custom algorithms or software that are central to the research but not yet described in published literature, software must be made available to editors and reviewers. We strongly encourage code deposition in a community repository (e.g. GitHub). See the Nature Portfolio guidelines for submitting code & software for further information.

Data

Policy information about availability of data

All manuscripts must include a data availability statement. This statement should provide the following information, where applicable:

- Accession codes, unique identifiers, or web links for publicly available datasets
- A description of any restrictions on data availability
- For clinical datasets or third party data, please ensure that the statement adheres to our policy

The authors declare that all data supporting the findings of this study are available within the article and its Supplementary Information files. Source data are provided with this paper.

Research involving human participants, their data, or biological material

Policy information about studies with human participants or human data. See also policy information about sex, gender (identity/presentation), and sexual orientation and race, ethnicity and racism.

Reporting on sex and gender	Not applicable
Reporting on race, ethnicity, or other socially relevant groupings	Not applicable
Population characteristics	Not applicable
Recruitment	Not applicable
Ethics oversight	Not applicable

Note that full information on the approval of the study protocol must also be provided in the manuscript.

Field-specific reporting

Please select the one below that is the best fit for your research. If you are not sure, read the appropriate sections before making your selection.

[x] Life sciences [ ] Behavioural & social sciences [ ] Ecological, evolutionary & environmental sciences

For a reference copy of the document with all sections, see nature.com/documents/nr-reporting-summary-flat.pdf

Life sciences study design

All studies must disclose on these points even when the disclosure is negative.

Sample size	In rat calvarial defects, we repeated multiple rounds of experiments to examine and confirm the therapeutic effect of sp-EMS. At each round of rat experiments, we included at least 5 rats in each group based on previously published priori power analyses(Adv Funct Mater 29, 1806445; Adv Healthc Mater 5, 1505-1512). Power analysis requires prior knowledge of two key parameters: (1) the effect size, defined as the minimum difference between groups considered clinically significant, and (2) the standard deviation, which measures variability within a sample for a quantitative variable. Without estimates of the effect size and standard deviation from previous similar studies, power analysis cannot be performed. Since this study is the first of its kind conducted in rabbits and dogs, it is impossible to assume the effect size and the standard deviation required for power analysis. For this reason, an alternative method, the 'resource equation' approach, which sets the acceptable range of degrees of freedom (DFs)(Malays J Med Sci 24, 101-105), was used to determine the sample size. To achieve a DF between 10 and 20, the minimum number for each group was 2.67, whereas the maximum number for each group was 4.33(Sci Transl Med 14, eabi7282). Therefore, n = 3 was used for each group in the in vivo study of the rabbits and beagle dogs, which follows the principles of the 3Rs (Reduction, Refinement, and Replacement) in the calculation of the sample size in animals.
Data exclusions	Because animals are gated, rats, rabbits and beagle dogs were excluded for poor body condition (e.g., weight loss, serious injuries and infections). Exclusion criteria were pre-established before the study. One broken tooth root in rabbits was excluded due to accidental breakage. No rats nor beagle dogs were excluded from the analysis.
Replication	At least three biological independent samples were performed for each experiment. All replications were successful.
Randomization	Samples were randomly assigned to experimental or control groups.
Blinding	All the surgical treatments and the functional experiments were done by blinded investigators.

Reporting for specific materials, systems and methods

We require information from authors about some types of materials, experimental systems and methods used in many studies. Here, indicate whether each material, system or method listed is relevant to your study. If you are not sure if a list item applies to your research, read the appropriate section before selecting a response.

Materials & experimental systems

n/a	Involved in the study
☐	☒ Antibodies
☐	☒ Eukaryotic cell lines
☒	☐ Palaeontology and archaeology
☐	☒ Animals and other organisms
☒	☐ Clinical data
☒	☐ Dual use research of concern
☒	☐ Plants

Methods

n/a	Involved in the study
☒	☐ ChIP-seq
☒	☐ Flow cytometry
☒	☐ MRI-based neuroimaging

Antibodies

Antibodies used

Antibodies used in Western Blot: anti-BMP-2 (1:1000, diluted with 5% w/v skim milk, Abcam, Cat#AB214821); anti-p-Smad1/5/9 (1:1000, diluted with 5% w/v skim milk, CST, Cat#13820); anti-t-Smad5 (1:1000, diluted with 5% w/v skim milk, CST, Cat#12534); anti-Actin (1:1000, diluted with 5% w/v skim milk, ZSGB-BIO, Cat#TA-09); anti-GAPDH (1:1000, diluted with 5% w/v skim milk, Proteintech, Cat#60004-1-lg); anti-F-Actin (1:1000, diluted with 5% w/v skim milk, Cytoskeleton, Cat#BK037); Secondary antibodies HRP-labeled (1:10000, diluted with TBST, ZSGB-BIO, Cat#ZB-2301 and Cat#ZB-2305).
Antibodies used in immunofluorescent staining: anti-CACNA2D1 (1:200, diluted with 3% w/v BSA, Abcam, Cat#AB238110); anti-TNF-α (1:200, diluted with 3% w/v BSA, Abcam, Cat#AB183218); anti-iNOS (1:200, diluted with 3% w/v BSA, Abcam, Cat#AB178945); anti-CD68 (1:200, diluted with 3% w/v BSA, Proteintech, Cat#28058-1-AP); anti-CD163(1:200, diluted with 3% w/v BSA, Abcam, Cat#AB156769); FITC-labeled goat anti-rabbit IgG(H+L) (1:200, diluted with PBS, ZSGB-BIO, Cat#ZF-0311); Rhodamine-labeled goat anti-mouse IgG(H+L) (1:200, diluted with PBS, ZSGB-BIO, Cat#ZF-0313).
Antibodies used in immunohistochemistry staining: anti-BMP-2 (1:200, diluted with 3% w/v BSA, Abcam, Cat#AB214821); anti-OCN (1:200, diluted with 3% w/v BSA, Abcam, Cat# AB93876); anti-VEGFR-1 (1:200, diluted with 3% w/v BSA, Abcam, Cat# AB2350); anti-CD31 (1:200, diluted with 3% w/v BSA, Abcam, Cat#AB32457); Horseradish enzyme labeled goat anti-rabbit IgG (1:200, diluted with PBS, ZSGB-BIO, Cat#PV-9001)

Validation

Anti-CACNA2D1: ab238110, key features and details: Rabbit polyclonal to Calcium channel L type DHPR alpha 2 subunit/CACNA2D1; Suitable for ICC/IF, IHC-P; https://www.abcam.cn/products/primary-antibodies/calcium-channel-l-type-dhpr-alpha-2-subunitcacna2d1-antibody-ab238110.html
Anti-BMP-2: ab214821, key features and details: Produced recombinantly (animal-free) for high batch-to-batch consistency and long term security of supply; Rabbit monoclonal [EPR20807] to BMP2; Suitable for Flow Cyt (Intra), WB, ICC/IF; https://www.abcam.cn/products/primary-antibodies/bmp2-antibody-epr20807-ab214821.html
Anti-OCN: ab93876, key features and details: Rabbit polyclonal to Osteocalcin; Suitable for WB, IHC-P; https://www.abcam.cn/products/primary-antibodies/osteocalcin-antibody-ab93876.html
Anti-VEGFR-1: ab2350, key features and details: Rabbit polyclonal to VEGF Receptor 1; Suitable for WB, IHC-P, ICC/IF, Electron Microscopy; https://www.abcam.cn/products/primary-antibodies/vegf-receptor-1-antibody-ab2350.html
Anti-CD31: ab32457, key features and details: Rabbit polyclonal to CD31; Suitable for ICC, IHC-P, WB, Flow Cyt (Intra); https://www.abcam.cn/products/primary-antibodies/cd31-antibody-ab32457.html
Anti-TNF-α: ab183218, key features and details: Produced recombinantly (animal-free) for high batch-to-batch consistency and long term security of supply; Rabbit monoclonal [EPR19147] to TNF alpha; Suitable for WB, IP, ELISA, ICC/IF; https://www.abcam.cn/products/primary-antibodies/tnf-alpha-antibody-epr19147-ab183218.html
Anti-CD68: 28058-1-AP, Rabbit polyclonal antibody to CD68; Suitable for IF, IHC, WB, ELISA; https://www.ptgcn.com/products/Cd68-Antibody-28058-1-AP.htm
Anti-p-Smad1/5/9: Phospho-SMAD1 (Ser463/465)/ SMAD5 (Ser463/465)/ SMAD9 (Ser465/467) (D5B10) Rabbit mAb #13820; Suitable for WB; https://www.cellsignal.cn/products/primary-antibodies/phospho-smad1- ser463-465-smad5-ser463-465-smad9-ser465-467-d5b10-rabbit-mab/13820?site-search type = Products & N = 4294956287 & Ntt = 13820&fromPage=plp&_requestid=2683998
Anti-Smad5: SMAD5 (D4G2) Rabbit mAb #12534; Suitable for WB; https: www. cellsignal.cn/products/primary-antibodies/smad5-d4g2-rabbit-mab/12534?site-search-type = Products & N = 4294956287 & Ntt = 12534&fromPage=plp&_requestid=2684684
Anti-CD163: ab156769, key features and details: Mouse monoclonal [OTI2G12] to CD163; Suitable for WB, IHC-P, ICC/IF; https://www.abcam.cn/products/primary-antibodies/cd163-antibody-oti2g12-ab 1567 69.html
Anti-iNOS: ab178945, key features and details: Mouse monoclonal [NOS-IN] to iNOS; Suitable for ICC/IF, WB; Reacts with: Mouse; Isotype: IgG1. https://www.abcam.cn/products/primary-antibodies/inos-antibody-nos-in-ab49999.html
Anti-GAPDH: 60004-1-Ig, Mouse monoclonal Antibody to GAPDH; Suitable for IF, IP, WB, ELISA; https://www.ptgcn.com/products/GAPDH-Antibody-60004-1-Ig.htm
Anti-actin: TA-09, Mouse monoclonal Antibody to β-Actin; Suitable for WB; http://www.zsbio.com/product/TA-09

Eukaryotic cell lines

Policy information about cell lines and Sex and Gender in Research

Cell line source(s)

Human BMSCs (PCS-500-012) were purchased from Beijing Zhongyuan Company Limited (Beijing, China).

Authentication

Each cell line used was morphologically confirmed according to the information provided by culture collections.

Mycoplasma contamination	All cell lines were negative for mycoplasma.
Commonly misidentified lines (See ICLAC register)	No commonly misidentified cell lines were used in the study.

Animals and other research organisms

Policy information about studies involving animals; ARRIVE guidelines recommended for reporting animal research, and Sex and Gender in Research

Laboratory animals	Male Sprague Dawley adult rats (6-8 weeks) were obtained from Weitong Lihua Experimental Animal Center (China). Adult female New Zealand rabbits with an average body weight of 2-2.5 kg (2.5-3 months old) and adult male beagle dogs (8 −10 kg, 1-1.5 years old) were also obtained from Weitong Lihua Experimental Animal Center (China) .Temperature(23±2°C) and humidity(55%) were held constant in animal housing.
Wild animals	No wild animals were used.
Reporting on sex	In order to exclude gender variable factors in animal testing, single-sex experimental animals were used in this study.
Field-collected samples	No samples collected from the field were used.
Ethics oversight	All experimental procedures used in this study were performed in compliance with animal welfare ethical regulations and approved by the Animal Use and Care Committee of Peking University (LA2022190).

Note that full information on the approval of the study protocol must also be provided in the manuscript.
